# Bridging disciplines: evaluating collaborative skills in interprofessional education between dentistry and pharmacy students “Collaborative skills evaluation in dentistry-pharmacy IPE”

**DOI:** 10.1186/s12909-025-07727-1

**Published:** 2025-08-12

**Authors:** Nouran Omar El Said, Marwa Ali-Tammam, Sherif Abdelrahman Amer, Eman Mohamed Elmokadem

**Affiliations:** 1 Department of Pharmacy Practice and Clinical Pharmacy, Faculty of Pharmacy, Future University in Egypt, 90th Street, New Cairo, 12311 Cairo Egypt; 2 Department of Microbiology and Immunology, Faculty of Pharmacy, Future University in Egypt, Cairo, Egypt; 3 Department of Oral Medicine, Diagnosis and Periodontology, Faculty of Oral and Dental Medicine, Future University in Egypt, Cairo, Egypt

**Keywords:** Interprofessional education, Team collaboration, Pharmacy, Competencies, Dentistry

## Abstract

**Background:**

Interprofessional education (IPE) is a crucial strategy for preparing pharmacy and dentistry students for the health workforce, focusing on teamwork and collaboration. It provides an integrated knowledge application between both fields to improve patient care in areas such as infection control, antimicrobial management, and pain management. This study aimed to examine the self-reported perceptions of pharmacy and dental students regarding their interprofessional collaborative competencies before and after participating in a pilot interprofessional education activity.

**Methods:**

An IPE activity was conducted from September 4–8, 2022, at Future University in Egypt (FUE), involving 26 students (19 from pharmacy and 7 from dentistry). Students were assigned to intercollaborative teams with student members from both schools. Sessions were held, and group discussions were utilized, punctuated by small group cases and simulation activities, to create interprofessional understanding. Facilitators consisted of faculty from both pharmacy and dentistry. Dental rounds at FUE Teaching Dental Hospital simulated interprofessional practice among team members. To assess the perceived skills acquired from the activity, participants completed a pre-and post-activity survey using the Interprofessional Collaborative Competencies Attainment Survey (ICCAS)– tool.

**Results:**

All 26 participating students completed the pre- and post-activity ICCAS survey. The students were cooperative during the overall experience and developed communication and interprofessional skills. The findings of the pre- and post-activity ICCAS yielded positive changes on all subscales. Significant differences (*p* < 0.05) were seen in all subscales when comparing pre-and post-activity scores using a Wilcoxon signed rank test.

**Conclusion:**

This IPE initiative increased collaboration and understanding between pharmacy and dentistry students. The improvements noted in interprofessional competencies emphasize the significance of such initiatives in equipping future health practitioners with skills necessary for collaborative work environments. It is recommended to continue and expand initiatives focusing on IPE to foster interprofessional cooperation, effective communication, and teamwork skills for optimal patient outcomes.

**Clinical trial number:**

not applicable.

## Background

Modern healthcare is complicated by chronic illness and complex therapies, and fueled by an aging population, no single healthcare professional can manage therapy alone [1, 2]. Therefore, interprofessional collaboration in healthcare is crucial to improve patient care.

For effective interprofessional collaboration in healthcare, interprofessional education (IPE) is recommended to be integrated into undergraduate, graduate, and pre-doctoral programs [3]. The World Health Organization (WHO) defines IPE as a process by which students of various health professions learn together, from one another, and about each other to improve their teamwork skills and thus create better health outcomes [4].

IPE advocates for participatory educational models to enhance shared learning encounters among the various health professions. Enhanced collaboration among the different health professions, leads to safer and higher-quality care while reducing demand on the healthcare system [5]. By merging diverse professional skills and resources, IPE helps health teams better meet their patients’ needs. To optimize teamwork and improve patient outcomes, learning from, with, and about each other is essential [2].

Lack of proper collaboration may arise when different healthcare professionals are not taught how to work as a unit towards common objectives. Interprofessional interactions should, therefore, be embedded in educational programs by including team-building activities at the undergraduate level [6]. Pertinently, integrating IPE in undergraduate and postgraduate curricula is crucial. This is important, bearing in mind that complexities in the health sector continue to rise, requiring healthcare providers of the future to have effective roles in multi-disciplinary teams.

IPE activities involving practice simulations and experiential learning opportunities are effective methods for immersing students in team-based environments [7]. This collaborative approach is particularly relevant for pharmacy and dentistry students, who, despite their distinct disciplines, frequently engage in overlapping areas of patient care and treatment planning, in which patient care can be enhanced through interprofessional collaborations [8].

Poor oral health due to untreated oral infections and inflammation can impact general health meaning dentists play an important role in a patient’s healthcare team [9]. Additionally, because dental professionals have a profound impact on a patient’s well-being, there is a need to promote proper collaboration between dentists and other healthcare providers to promote public health [10]. The recent technological advancements including new biomedical technologies or applied genomics indicate that dental professionals must partner with their colleagues in different specialties to provide comprehensive care [11].

The involvement of pharmacists in recognizing and resolving drug-related problems during initial patient visits is vital as well as assisting timely referrals to dental care providers when necessary [12]. They manage systemic conditions that can result in avoidable hospital admissions if oral symptoms are poorly controlled. Moreover, medication-related oral health problems like oral fungal infections or oral dehydration may be handled by pharmacists. They could also help dentists by identifying patients with a high potential for poor oral health and advising about drug interactions while promoting oral hygiene practices [11]. The collaboration between dentists and pharmacists can decrease individual burdens and help enhance patient care outcomes [13].

IPE activities involving students from both pharmacy and dentistry programs promoted efficient teamwork among future health professionals [14]. This form of learning relies on active engagement and support from cohorts of learners, trainers, or associations based on different teaching methods to improve education levels [13]. Assessing students’ performance before and after such programs provides a further understanding of IPE capabilities for enhancing collaboration, interprofessional engagement, and future practice [15].

Research has indicated that students who have undergone IPE tend to have positive attitudes toward collaboration, while those who have not received it do not [6]. Despite the availability of IPE in numerous medical programs, joint IPE activities involving dentistry or pharmacy programs are scarce. The study aimed to provide information on the effectiveness of a pilot-implemented IPE experience by examining how participants perceive their competency skills and readiness for collaboration. Recognizing these perceptions will help improve instructional methods and ensure that tomorrow’s health workers are equipped with the abilities necessary to function in a team-based atmosphere.

## Methodology

### Participants and setting

Egyptian undergraduate programs in dentistry and pharmacy are rigorous five-year curricula concluding in bachelor’s degrees that qualify graduates for professional practice. These programs emphasize discipline-specific knowledge and clinical skills within their respective fields. Interprofessional education (IPE) was not part of the core curriculum for either discipline, with students traditionally learning within their professional roles.

This IPE activity was conducted as a pilot experience where participation was voluntary. After announcing the week-long activity to senior students (in their 4th or 5th year at the Future University in Egypt (FUE)) from both pharmacy and dental schools, interested students could apply to participate. Only participants willing to engage in the study were further requested to complete the study survey tool. All included participants provided informed consent, acknowledging their understanding of the study’s objectives and scope.

### Interprofessional educational Experience- activities description

The IPE was comprehensively designed through the collaboration between faculty members from the Faculty of Pharmacy and the Faculty of Oral and Dental Medicine, with guidance from the IPE coordinator at the Faculty of Pharmacy. This collaborative approach to designing the activities ensured that relevant learning objectives from both disciplines were appropriately integrated, with careful attention to competencies or capabilities for IPE. Faculty facilitators from both pharmacy and dentistry participated in planning sessions to ensure consistent delivery of content and alignment with the four IPEC core competencies.

The four core competencies for IPE as defined by the Interprofessional Education Collaborative (IPEC) are values/ethics for interprofessional practice, roles/responsibilities, interprofessional communication, and teams and teamwork [16]. This allowed a multi-modal learning experience concerning professional roles, teamwork, case-based simulations, and clinical rounds regarding practical exposure. The multi-modal experience encouraged students to become familiar with their colleagues’ disciplines, work in mixed teams, experience real-life scenarios, and apply their skills in clinical settings under supervision. Such a structure laid a solid foundation for building interprofessional cooperation and understanding.

The IPE activity was conducted over five consecutive days during which the students remained in their same designated teams. Day 1 focused on team formation through icebreaker activities, interactive learning about healthcare roles, and the introduction of the hospital setting and patient filing. Days 2–4 followed a consistent structure: daily team gatherings for case simulations, followed by clinical rounds in the Dental hospital and concluding with reflection sessions. The final day (Day 5) included specialized knowledge exchange sessions covering oral microbiota and drug-induced oral disorders, concluding in a final reflection session and completion of the ICCAS survey. The description of the activities is detailed below.

#### Interactive learning on healthcare roles

Students were purposefully placed in heterogeneous teams that included pharmacy and dentistry students to promote interdisciplinary collaboration and enhance their skills through teamwork and communication for healthcare roles, in related fields. Facilitators consisted of faculty from both pharmacy and dentistry.

The session started with icebreaker games to build relationships among students within the teams. This was followed by reflective activities encouraging them to think about and discuss the roles that healthcare professionals play in a team setting. The goal of the reflective activity was to set a solid foundation for further exploration into the different aspects of interprofessional collaborative practice competency areas.

#### Case-Based collaborative learning

The core of the activity was centered on case-based learning activities. Student teams collaborated on four different problem-solving case scenarios intended to merge knowledge of pharmacy and dentistry. The cases were collaboratively developed by experienced faculty members from the Faculty of Pharmacy and Oral and Dental Medicine. The main activities included joint medical history-taking exercises, stressing the importance of collecting comprehensive data from various perspectives in healthcare. The case-based exercise focused on core areas where pharmacy overlaps with dentistry, such as the selection of antibiotics for dental infections, formulation of multimodal pain management plans, local anesthesia considerations during dental procedures, and drug-interaction identification and management among dental patients. The intention was to show how pharmacy complements dentistry in patient care through these activities.

#### Clinical exposure: hospital rounds

The collaborative rounds at the Teaching Dental Hospital were a pivotal aspect of the activity. This hands-on experience enabled inter-professional student groups to interview real patients, thus acquiring communication skills and learning how to collect adequate information considering both dental and pharmaceutical perspectives. Additionally, they analyzed patient files with an emphasis on comprehensive documentation and the sharing of information among healthcare practitioners. These rounds further helped identify drug therapy-related problems and shared discussions utilizing the combined knowledge of students from pharmacy and dentistry to formulate strategies for comprehensive care. The rounds were facilitated by staff from both schools.

#### Specialized knowledge exchange

To further enhance interprofessional learning, focused educational sessions on topics that connect pharmacy with dentistry were part of the activity. These included an overview of oral microbiota as it applies to dental and general health. Furthermore, a thorough discussion of medication-induced oral disorders was delivered, highlighting the need for pharmacological knowledge in dental practice and the need to take oral health into account when administering medications. This enforced the awareness of the various contributions made by each profession to healthcare while supporting the idea that the two areas are interconnected in patient care. Throughout the sessions, facilitators from the pharmacy and dentistry faculties emphasized the core competencies of interprofessional collaborative practice [16]. Students were encouraged to reflect on their experiences, discussing how the activities enhanced their understanding of team-based healthcare delivery and the value of interdisciplinary cooperation in improving patient outcomes.

This structured approach to IPE aimed to provide students with practical experience in collaborative care, enhance their understanding of other healthcare roles, and prepare them for effective teamwork in their future professional practice.

### Ethical considerations

#### Informed consent

had been obtained from all study participants. The entire study procedure followed the standards of Good Clinical Practice and the Helsinki Declaration for Human Subjects. The Faculty of Pharmacy at Future University in Egypt’s Research Ethics Committee approved this study. Before taking part, participants received comprehensive information on the goals and methods of the study. Their responses were only recorded and incorporated into the analysis after they had confirmed their comprehension and provided their consent. Participants’ anonymity was rigorously preserved, and they were assured that their answers would remain confidential.

### Survey instrument & data collection

To measure changes in participants’ skills due to this IPE activity, the Interprofessional Collaborative Competencies Attainment Survey (ICCAS) was administered after completing the IPE to rate their competencies pre- and post-activity, retrospectively [17]. The ICCAS is tailored for assessing multiple dimensions of collaborative competencies by evaluating self-reported attainment of skills related to interprofessional communication, collaboration, team member roles and responsibilities, collaborative patient/family-centered approach, conflict management/resolution, and team functioning. Based on a 7-point Likert-type scale, participants rated their agreement with 20 statements that provided insights into their perceived competencies before and after the activity. Baseline levels of cooperative skills were established using the pre-activity questionnaire. Any alterations in these abilities arising out of the IPE experience were recorded through the post-activity survey.

Satisfaction data was collected using a structured 5-point Likert scale (highly satisfied, satisfied, neutral, unsatisfied, highly unsatisfied). Open-ended feedback was collected through written responses at the conclusion of the IPE experience, allowing students to freely express their experiences and perceptions.

### Data analysis

We analyzed the survey data to identify significant changes in the participants’ self-reported competencies. Descriptive statistics summarized the survey responses, and Wilcoxon signed ranks were used to compare pre-and post-activity scores. Statistical significance was set at *p* < 0.05. The effect size (r) was calculated and interpreted as a large effect size if > 0.5, a moderate effect size if > 0.3, and a small effect size if > 0.1. No effect or negligible effect was considered if < 0.1. This analysis helped to determine the effectiveness of the IPE activity in improving collaborative skills.

## Results

The study was conducted from September 4th to 8th, 2022 at Future University in Egypt. The original class sizes were approximately 300 students in each discipline (dentistry and pharmacy). However, this interprofessional activity was conducted as a voluntary pilot experience and was not integrated into the formal curriculum. A total of 26 students, from the Faculty of p(*N* = 19) and the Faculty of Oral and Dental Medicine (*N* = 7) participated in the IPE activity. The facilitator-to-student ratio was 1:6.5. All students who agreed to participate in the study responded to a pre-and post-activity survey with a 100% response rate of the survey collected. The pre-and post-activity scores for each subscale are summarized in Table 1. Furthermore, we assessed the effect sizes of all the subscales of the ICCAS as illustrated in Table 2.

**Table 1:** Changes in ICCAS score

	Pharmacy students’ responses (*n* = 19)		*p*- value ^a^	Dentistry students’ responses (*n* = 7)				*p*- value ^a^	All participants’ responses (*n* = 26)		*p*- value ^a^
	Pre- activity Median (IQR)	Post-activity Median (IQR)		Pre- activity Median (IQR)		Post-activity Median (IQR)			Pre- activity Median (IQR)	Post-activity Median (IQR)	
**Communication**											
1. Promote effective communication among members of an interprofessional (IP) team	5(4–5)	6(5–6)	< 0.001	5(4–6)	5(5–6)		0.046		5(4–5)	6(5–6)	< 0.001
2. Actively listen to IP team members’ ideas and concerns	5(4–6)	5(5–7)	0.002	5(4–6)	5(5–7)		0.046		5(4–6)	5(5–7)	< 0.001
3. Express my ideas and concerns without being judgmental	5(4–5)	5(5–6)	< 0.001	5(4–5)	5(5–6)		0.025		5(4–5)	5(5–6)	< 0.001
4. Provide constructive feedback to IP team members	5(5–6)	6(5–6)	0.001	5(4–5)	6(5–6)		0.020		5(4–5)	6(5–6)	< 0.001
5. Express my ideas and concerns in a clear, concise manner	5(4–5)	5(5–6)	0.001	4(4–5)	5(5–6)		0.020		4(4–5)	5(5–6)	< 0.001
**Collaboration**											
6. Seek out IP team members to address issues	5(4–5)	5(4–6)	0.021	4(4–5)	5(5–6)		0.020		5(4–5)	5(4–6)	0.002
7. Work effectively with IP team members to enhance care	4(5 − 4)	5(5–6)	0.002	4(4–5)	5(5–6)		0.034		5(4–5)	5(5–6)	< 0.001
8. Learn with, from and about IP team members to enhance care	5(4–6)	5(5–6)	0.002	4(4–5)	5(5–6)		0.034		5(4–5)	5(5–6)	0.004
**Roles and Responsibilities**											
9. Identify and describe my abilities and contributions to the IP team	5(4–5)	5(5–6)	0.002	5(5–5)	5(5–6)		0.049		5(4–5)	5(5–6)	0.002
10. Be accountable for my contributions to the IP team	5(5–5)	5(5–6)	0.008	5(4–5)	6(5–6)		0.034		5(4–5)	5(5–6)	< 0.001
11. Understand the abilities and contributions of IP team members	5(4–5)	5(5–6)	0.001	4(4–5)	5(5–6)		0.034		4(4–5)	5(5–6)	< 0.001
12. Recognize how others’ skills and knowledge complement and overlap with my own	5(4–5)	5(5–6)	0.023	4(4–5)	5(5–6)		0.039		5(4–5)	5(5–6)	< 0.001
**Collaborative Patient/Family-Centred Approach**											
13. Use an IP team approach with the patient to assess the health situation	5(4–5)	5(4–6)	0.008	5(4–5)	5(5–6)		0.043		5(4–5)	5(5–6)	0.004
14. Use an IP team approach with the patient to provide whole person care	5(4–5)	5(5–6)	0.003	5(4–5)	5(5–7)		0.025		5(4–5)	5(5–6)	< 0.001
15. Include the patient/family in decision-making	5(4–5)	5(5–6)	0.001	5(4–5)	6(5–6)		0.038		5(4–5)	5(5–6)	< 0.001
**Conflict Management/Resolution**											
16. Actively listen to the perspectives of IP team members	5(4–5)	5(5–6)	0.002	4(4–5)	5(5–6)		0.02		5(4–5)	5(5–6)	0.004
17. Take into account the ideas of IP team members	5(4–5)	5(5–6)	0.023	4(4–5)	5(4–6)		0.034		5(4–5)	5(5–6)	< 0.001
18. Address team conflict in a respectful manner	5(4–5)	5(5–6)	0.002	4(4–5)	5(5–6)		0.059		5(4–5)	5(5–6)	< 0.001
**Functioning**											
19. Develop an effective care plan with IP team members	5(4–5)	5(4–6)	0.005	5(4–5)	5(5–6)		0.025		5(4–5)	5(4–6)	< 0.001
20. Negotiate responsibilities within overlapping scopes of practice	4(4–5)	5(4–5)	0.048	4(4–5)	5(5–6)		0.034		4(4–5)	5(4–5)	0.005

^**a**^ Wilcoxon signed rank, p-value < 0.05, Statistical significance: *p* < 0.05

IP: interprofessional

Pre- and post-scores were based on a 7-point Likert scale: 1 = strongly disagree; 2 = moderately disagree; 3 = slightly disagree; 4 = neutral; 5 = slightly agree; 6 = moderately agree; 7 = strongly agree

**Table 2:** Effect size of IPE activity

	Pharmacy students’ responses (*n* = 19)	Dentistry students’ responses (*n* = 7)	All participants’ responses (*n* = 26)
	Effect size (*r*)^a^	Effect size (*r*) ^a^	Effect size (*r*) ^a^
**Communication**			
1. Promote effective communication among members of an interprofessional (IP) team	0.59	0.53	0.57
2. Actively listen to IP team members’ ideas and concerns	0.51	0.54	0.51
3. Express my ideas and concerns without being judgmental	0.56	0.59	0.57
4. Provide constructive feedback to IP team members	0.52	0.62	0.54
5. Express my ideas and concerns in a clear, concise manner	0.54	0.63	0.56
**Collaboration**			
6. Seek out IP team members to address issues	0.37	0.62	0.45
7. Work effectively with IP team members to enhance care	0.51	0.57	0.52
8. Learn with, from and about IP team members to enhance care	0.49	0.56	0.51
**Roles and Responsibilities**			
9. Identify and describe my abilities and contributions to the IP team	0.49	0.48	0.49
10. Be accountable for my contributions to the IP team	0.43	0.57	0.46
11. Understand the abilities and contributions of IP team members	0.52	0.56	0.53
12. Recognize how others’ skills and knowledge complement and overlap with my own	0.52	0.62	0.545
**Collaborative Patient/Family-Centred Approach**			
13. Use an IP team approach with the patient to assess the health situation	0.42	0.53	0.49
14. Use an IP team approach with the patient to provide whole person care	0.48	0.55	0.50
15. Include the patient/family in decision-making	0.36	0.50	0.40
**Conflict Management/Resolution**			
16. Actively listen to the perspectives of IP team members	0.51	0.57	0.52
17. Take into account the ideas of IP team members	0.37	0.51	0.40
18. Address team conflict in a respectful manner	0.30	0.43	0.39
**Functioning**			
19. Develop an effective care plan with IP team members	0.46	0.59	0.50
20. Negotiate responsibilities within overlapping scopes of practice	0.49	0.50	0.49

^**a**^ Wilcoxon signed rank

IP: interprofessional

The baseline (pre-activity) ICCAS scores were positive in all the competency categories for students from both disciplines. The analysis of the data from the ICCAS survey revealed significant improvements in self-reported competencies across all 20 subscales after the IPE activity for the total participants from both faculties. The same was observed in the ICCAS scores of pharmacy students alone. When analyzing pre- and post-ICASS scores in dentistry students alone, most of the scores indicated significant improvement, except in domain 18: “address team conflict in a respectful manner”.

The items that reported the smallest effect sizes in pharmacy students were domain 15: “include the patient/family in decision-making” (*r* = 0.36) and domain 18: “address team conflict in a respectful manner” (*r* = 0.30). Dentistry students reported the smallest effect sizes in domain 18: “address team conflict in a respectful manner” (*r* = 0.43) and domain 9: “identify and describe my abilities and contributions to the IP team” (*r* = 0.48). The effect sizes ranged from moderate to large in all subscales across students from both disciplines.

Satisfaction rates were collected after completion of the IPE experience using a Likert scale. All of the participants from both faculties reported being satisfied with the activity (11[42%] highly satisfied; 15[58%] satisfied).

The IPE activity received positive open-ended feedback from many participating students. One of the undergraduate dental students commented “I never thought that the two professions would be so much related. It was interesting to cooperate with the students from pharmacy.” One of the pharmacy students expressed “It helped me appreciate the need for proper communication within the health professional’s context. I am now more comfortable working with dentists.” Another student said, “Well, the cases were not easy, but they brought out the message of the need for us all to come together.” Some students said that they enjoyed the practical components of the activities, one of them said, “Actually doing these exercises in the dental practice together made the biggest change, this is something you cannot read from the book.” The desire to connect and collaborate across the different types of study was also expressed, with one student stating “I have contacts which I think will be very useful once I start working.”

## Discussion

IPE aims to equip future healthcare professionals with the skills necessary for effective collaboration in multidisciplinary teams. This approach seeks to enhance mutual respect and understanding across the healthcare disciplines [2]. However, the effectiveness of such educational programs remains an issue under study, especially regarding the perceived competency levels in collaboration between pharmacy and dentistry students.

The implemented IPE involved collaboration between pharmacy and dentistry students through various educational methodologies. It was designed to include interactive group discussions, case-based simulation activities, and dental rounds at FUE Teaching Dental Hospital, allowing students to practice together in a clinical setting. As shown in Table 1, pre- and post-activity ICCAS scores revealed statistically significant improvements across all subscales (*p* < 0.05), indicating substantial improvement in students’ self-reported interprofessional competency skills. The effect sizes shown in Table 2 further support these findings, with moderate to large effects observed across nearly all competency domains, suggesting that the multi-modal approach adopted effectively promoted the development of essential inter-professional skills. However, no meaningful statistical comparison could be made between students’ responses in the different disciplines due to the unbalanced distribution of students.

The findings offer unique insights into IPE while validating previous research suggesting that implementing similar IPE activities can potentially increase collaborative abilities among pharmacy and dentistry learners.

Our study’s high baseline ICCAS scores may be attributed to the voluntary nature of the pilot activity. Students who chose to participate likely had a pre-existing interest in interprofessional collaboration and higher motivation to engage in IPE experiences. This self-selection may have resulted in a sample of participants who already possessed greater confidence in their interprofessional abilities prior to the intervention.

Our findings align with Williamson et al., who reported an IPE activity between pharmacy and dental hygiene students through case studies-based learning with standardized patient cases, virtual consultations, and self-reflective writing. Both studies found that despite high baseline levels of perceived competence, students demonstrated significantly improved ICCAS scores following the IPE activity. This finding suggests that even students who initially feel confident in their interprofessional abilities can benefit meaningfully from structured IPE experiences regardless of the delivery method [18].

Similarly, another report confirmed high baseline competency with significant improvement across self-reported domains. Despite cohort size imbalances between pharmacy and dentistry students in our study and the previous one, increased perceived interprofessional collaboration skills indicate successful implementation [19].

The significant improvements we observed across ICCAS subscales add to the conclusions reported in previous research by Pogge et al. regarding enhancing the understanding of dental pharmacotherapy. While the current study sought to assess the IPE participating group only on pre- and post-measures, their study included a non-participant control group showing similar positive outcomes in attitudes and knowledge. In this context, some of our qualitative feedback, such as “I never thought that the two professions would be so much related,” resonates with what they have called improvement in their understanding of disciplines [20].

The moderate to large effect sizes across all subscales for both dentistry and pharmacy students, shown in Table 2, likely reflect the effectiveness of our multi-modal approach. Combining role-play, simulation cases, clinical exposure, and scientific exchange in a team-based environment gave students diverse learning opportunities and learning styles that may have significantly enhanced their interprofessional competencies in different contexts. These findings support the uniqueness of the current study, unlike IPE experiences that typically used single or dual-modality approaches.

The most substantial improvements in the current study were seen in domains related to interprofessional communication and understanding of professional roles. Slightly smaller effect sizes were observed in conflict management (domain 18: “address team conflict in a respectful manner”) for both groups, particularly among dentistry students (*r* = 0.43). This reported detailed examination of domain-specific outcomes provides measurable evidence for how well IPE activities work. The smaller effect sizes observed in conflict management competencies highlight an opportunity for program enhancement. Future sessions could include more focused conflict resolution practice, including hands-on training in managing difficult conversations and defusing tense situations. Adding real examples of team conflicts and their solutions would give students practical strategies to use. These changes would help improve the weaker areas while maintaining the program’s overall effectiveness in other competency domains.

Our finding of smaller effect sizes in conflict management differs from the uniformly positive outcomes of the study by Theodorou et al. assessing early-stage, hands-on IPE in preparing pharmacy and dental students for collaborative practice. This difference might be attributed to our smaller sample size of dental students or variations in how the IPE activities addressed conflict management scenarios [21].

Our moderate-to-large effect sizes across subscales align with findings from Morelli et al., who analyzed an IPE activity involving 14 PharmD students in a Dental facility using Students’ Perceptions of Interprofessional Clinical Education Revised (SPICE-R2). Although they used a different assessment tool, along with diary entries and audio recordings, their results similarly showed positive statistical differences in mean scores for roles, responsibilities, and patient outcomes. This consistency in effect sizes across different measurement approaches strengthens the evidence for IPE effectiveness in pharmacy-dentistry collaboration [22].

Student qualitative feedback such as “Actually doing these exercises in the dental practice together made the biggest change, this is something you cannot read from the book” reinforces our quantitative findings on competency improvement. This is consistent with Branch-Mays et al.‘s research on teamwork effectiveness in handling drug therapy problems. Our interprofessional competency-based measurements complement their documented improvement in clinical outcomes, suggesting that practical clinical experiences make meaningful interprofessional skill development [23].

Our satisfaction data showed 100% positive responses (42% highly satisfied, 58% satisfied), providing quantitative support for the acceptability of our IPE approach. This agrees with the high satisfaction rates reported in previous studies [19, 21], though exact percentages varied due to different rating scales and sample sizes.

A key constraint in comparing our results with previous studies is the variation in assessment tools and statistical reporting methods. ICCAS measures the self-reported competencies of interprofessional collaboration pre- and post-activity, whereas SPICE looks at student perceptions of physician-pharmacist collaboration. A revised version, SPICE-R, has been developed to include other professions [24]. Each tool has different strengths and is selected depending on the particular goals and context of the IPE activity under assessment. ICCAS is a fully comprehensive assessment, while SPICE and SPICE-R are offered to determine insight into students’ attitudes. While all studies demonstrated positive outcomes, utilizing different metrics and varying sample sizes makes direct effect size comparisons challenging. Future research would benefit from standardized assessment approaches and reporting of effect sizes across all domains.

Team-based learning (TBL) has been highly implemented in pharmaceutical education [25, 26]. Though the current study did not adopt the typical TBL model, using it in a multi-disciplinary healthcare education environment enriches the learner’s understanding of long-term care concepts in new ways. This also helps develop critical professional skills like communication and teamwork [14]. Incorporating several educational experiences involving not just multidisciplinary but multicultural experiences in pharmaceutical education and diverse exposures to different healthcare practices may enhance students’ skills and ensure highly competent healthcare professionals [27].

The implementation phase of this IPE initiative revealed several operational challenges that required careful consideration, as commonly reported in the existing literature [8]. Scheduling conflicts between the two programs and possibly impacting participation and continuity was a big hurdle. Finding common ground for collaborative learning between the curricula of pharmacy and dentistry presented some problems because of the intrinsic differences in structure and focus of their curricula. Assessment of students from different healthcare disciplines proved complex, where each field has unique competency requirements and evaluation criteria. While direct assessment of interprofessional performance would be valuable, developing standardized evaluation criteria that fairly capture contributions from both pharmacy and dental students presented significant challenges. This restriction was addressed using the validated ICCAS tool, which measures self-perceived interprofessional competencies.

Moreover, logistic barriers and difficulties were encountered during the IPE implementation. There was difficulty in finding faculty members experienced in both fields to lead the IPE activities. Additionally, coordinating resources such as physical space, equipment, and materials across two faculties was complicated. Furthermore, a balance between the depth of content in pharmaceutical and dental aspects within a broad collaborative focus; needed careful planning and execution. A study addressed the most common implementation barriers in their IPE experience through video-conferencing and assigned roles, with significant improvements in interprofessional collaboration skills using minimal faculty resources [19]. These approaches may help offer valuable insights for enhancing our future IPE implementations.

Nevertheless, the current IPE between students of pharmacy and dentistry represents one important step toward furthering interprofessional collaboration in health education and doubtless will be refined and extended in future events.

### Study limitations, strengths, and future perspectives

There are several limitations in this study. Firstly, the sample size was quite small, consisting of students from just one institution, which may limit its generalizability. Furthermore, due to the short duration of the IPE activity, it may have lacked depth in collaborative skills development. A key limitation was the absence of instructor-based assessment of collaborative skills and competencies. While the ICCAS tool provided valuable insights into students’ self-perceived improvements, direct observation and assessments of interprofessional interactions by faculty members would have provided more objective evidence of skill development. Additional limitations include the unequal distribution of participants between disciplines (19 pharmacy vs. 7 dentistry students), which limits our ability to make meaningful statistical comparisons between groups. Furthermore, while qualitative feedback provided valuable context, our study was not designed as a formal mixed-methods study, which would have allowed for more systematic qualitative data collection and analysis.

Despite the small sample size, the reported pilot study limitation was compensated by a few advantages. The small cohort size proved beneficial for the pilot, as it allowed us to deliver a comprehensive educational experience because the number of students per facilitator was manageable, and student activities in the hospital were also facilitated. While the self-selected nature of participation and relatively small sample size should be considered when interpreting the results, the 100% response rate to pre- and post-activity surveys provides reliable data for this initial pilot implementation, eliminating any potential response bias within our participant group and enhancing internal validation. Furthermore, this arrangement allowed for more focused implementation and detailed observation of the interprofessional learning experience. A notable strength of our IPE initiative was its uncommon multi-modal approach combining didactic, simulation, and clinical experiences specifically for pharmacy and dental students. This approach across different learning environments likely enhanced student engagement and contributed to the positive outcomes observed.

For future research, it is suggested that a wider range of healthcare students from various disciplines and institutions be included in the participant pool. Future studies can utilize larger samples with more varied interprofessional activities to examine their in-depth effects. Additionally, longitudinal studies should be carried out to investigate the long-term impact of IPE on professional practice. Moreover, applying qualitative approaches in addition to summative evaluation may offer a deeper understanding of particular dimensions of teamwork that best support learning, and consideration of a mixed methods design to provide deeper insights into the mechanisms underlying competency improvements observed through quantitative measures. Future implementations could benefit from incorporating structured instructor evaluations alongside self-reported measures and formal facilitator training, and calibration protocols.

Henceforth, it would be appropriate to incorporate more IPE activities into the curriculum so that the continual development and application of collective competencies can take place among prospective health workers. Moreover, future IPE sessions should be designed to focus on the weaker areas identified in this pilot, particularly conflict management skills.

## Conclusion

Findings of the pre-and post-activity ICCAS survey showed an improvement in all self-reported interprofessional competency subscales. This suggests that the IPE activity successfully achieved its objective of enhancing the student’s skills in communication, teamwork, and understanding of professional roles. The results imply that students from the faculties of dentistry and pharmacy are recommended to further participate in such collaborative IPE experiences. Such events are essential to foster cooperative competence, which would, in turn, lead to improved outcomes within healthcare delivery systems.

## Data Availability

The datasets used and/or analysed during the current study are available from the corresponding author upon reasonable request.

## Abbreviations

Abbreviations: Definitions
IPE: Interprofessional education
IPEC: Interprofessional Education Collaborative
FUE: Future University in Egypt
ICCAS: Interprofessional Collaborative Competencies Attainment Survey
WHO: World Health Organization
IP: Interprofessional
SPICE: Students Perceptions of Interprofessional Clinical Education
SPICE-R: Students Perceptions of Interprofessional Clinical Education Revised
TBL: Team-Based Learning
